# Synthesis of a Diamino Substituted Terphenyldivinyl Chromophore

**DOI:** 10.3390/molecules14062111

**Published:** 2009-06-10

**Authors:** Zhen-Ting Du, Ru Liu, Jun-Ru Wang, An-Pai Li

**Affiliations:** 1 College of Sciences, Northwest A&F University, Yangling, Shaanxi 712100, China; E-mails: liuru75@gmail.com (R.L.), wangjr07@163.com (J-R.W.); 2 Synthetics Technologica Pte Ltd, 3 Phillip Street, #18-00 Commerce Point, 048693, Singapore;E-mail: anpaili@sina.com (A-P.L.)

**Keywords:** chromophore, energy transfer, synthesis, terphenyldivinyl

## Abstract

(*E,E*)-1,4-bis(4'-aminostyryl)-2,5-bis(octyloxy)-benzene (**6**) and its derivative (*E,E*)-1,4-bis(4'-acetamidostyryl)-2,5-bis(octyloxy)-benzene (**7**) were synthesized and characterized after alkylation, bromomethylation, Horner-Emmons reaction and reduction from hydroquinone. In order to gain more molecular electronic data, HOMO and LUMO of compound **6** have been calculated by Gaussian 03 W.

## Introduction

**Figure 1:**
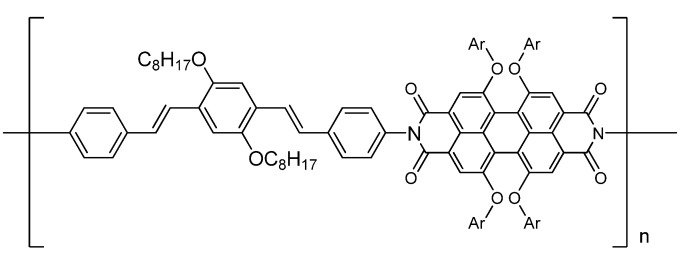
Designed polymer with terphenyldivinyl and perylene moiety.

As we all know, photosynthesis is the most important way on which all the lives in our planet depend directly or indirectly. Now, photosynthesis has been understood as a series sequence of energy and charge transfer in plants or microorganisms. Some photo-voltaic processes are designed based on mechanism of photosynthesis [1,2,3]. However, there is still a necessity to design and discover new chromophores to meet the need in research of charge or energy transfer. At the same time, organic light-emitting devices (OLED) employing organic chromophores as emitters have been the focuses of considerable interest because of their possible application as display for mobile phones, personal computers, and television [4,5]. Oligophenylvinyl [6,7,8,9] is a common chromophore used in study of energy transfer or in OLED. A diamino substituted oligophenylvinyl (*E,E*)-1,4-bis(4'-aminostyryl)-2,5-dimethoxybenzene (BDB) have been reported by Wu [10], but the synthetic process was not published. As shown in Figure 1**,** we have designed a polymer consist of terphenyldivinyl and perylene tetracarboxylic anhydride [11,12,13,14,15,16] which through double imide bond in our future research. 

Because of the low solubility of perylene moiety in common solvent, the long carbon chain was introduced. So oligophenylvinyl chromophore **6** and its acetylated derivative **7** were devised and synthesized. Herein, we describe the synthesis of these series chromophores and the results of theoretical computation.

## Results and Discussion

**Scheme 1:**
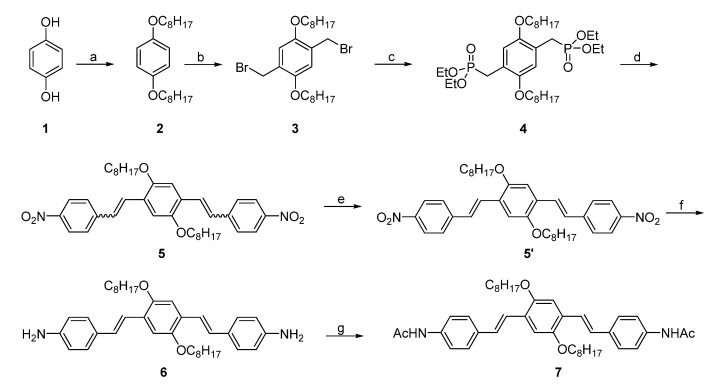
Synthesis of **6** and **7**.

As shown in Scheme 1, the long alkyl chain ether in the middle phenyl ring can increase the solubility of the chromophores in common solvent considerably, the terminal amino group is a versatile functional group to link with other chromophores, and meanwhile, the O and N substituted ones can elevate the HOMO level. 

*Reagents and conditions*: (a): KOH, *n*-C_8_H_17_Br, DMSO, reflux, 80^o^C, 75%; (b): 40% HBr, HOAc, (CH_2_O)_n_, reflux, 71%; (c): P(OEt)_3_, reflux; (d) *t*-BuOK, 4-nitrobenzyladehyde, THF, 74% for two steps; (e) I_2_(cat.), Tol., reflux, 95%; (f) SnCl_2_^.^2H_2_O, EtOH, EtOAc, 83%; (g): AcCl, Et_3_N, DMAP, 96%.

The synthetic strategy employed for the synthesis of **7** was based on the Horner-Emmons reaction. Hydroquinone was used as starting material, which was alkylated in DMSO with C_8_H_17_Br in the presence of KOH, and then it was subsequently treated with conc. HBr and paraformaldehyde, to give a benzyl bromide derivative. Compound **3** was reacted with P(OEt)_3_ to give the phosphonate **4**. After reaction of 4-nitrobenzyladehyde and the phosphonate **4**, a mixture of *cis* and *trans* isomers was obtained. In order to get all *trans* product, a catalytic isomerization of the mixtures was performed in the presence of iodide in toluene to convert the *cis* isomers to compound **5’**. A tin (II) chloride reduction of the nitro groups [17] afforded diamino substituted terphenyldivinyl derivative **6**. Compound **6** was subjected to a conventional acetylation process to give compound **7**.

**Figure 2:**
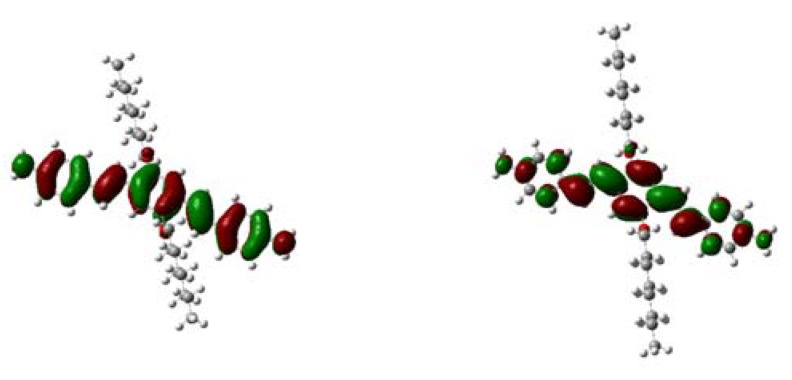
HOMO and LUMO of **6** at the B3LYP/6-31G.

Moreover, to gain insight into the electronic properties of our target molecular, its molecular geometry was fully optimized at the B3LYP/6-31G level 10 using Gaussian 03 package. As shown in Figure 2, the contour of HOMO and LUMO of compound **6** was given after computational calculation. The HOMO and LUMO were condensed mostly on middle benzene ring, and the long alkyl chains were distributed symmetrically. The levels of HOMO and LUMO were -4.41eV and -1.20eV, respectively. 

The absorption and emission spectrum were also evaluated. The absorption peak of compound **6** and **7** are at 401 nm and 397 nm, and the max emission are at 466 nm and 458 nm, respectively. This emission wavelength indicated that it can be cooperated with perylene moiety as donor [16].

**Figure 3:**
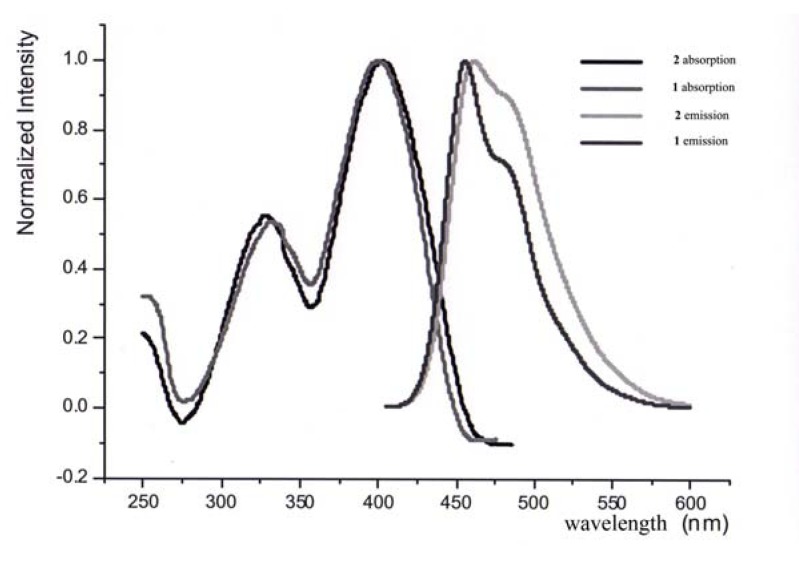
The absorption and emission of **6** and **7** in CH^2^Cl^2^.

## Experimental

### General

The ^1^H-NMR and ^13^C-NMR data were recorded in CDCl_3_ solution with Bruker AM-200 or AM-400 MHz spectrometers. The chemical shifts are reported in ppm relative to TMS or CDCl_3_. Column chromatography were generally performed on silica gel (200-300 mesh) eluting with petroleum ether:EtOAc (100:1-10:1 v/v) and TLC inspections on silica gel GF254 plates with petroleum ether:EtOAc ( 20:1-5:1 v/v ) if not noted otherwise.

*1,4-bis(Octyloxy)benzene* (**2**): Under Ar, a mixture of hydroquinone (55.0 g, 0.5 mol), KOH (67.2 g, 1.2 mol) and octyl bromide (251.0 g, 1.3 mol) in DMSO (400 mL) was stirred for 48 hours at 80^o^C. after the reaction completion and cooling, the reaction mixture was poured into water (2000 mL) with stirring, the precipitate was collected and washed with water (300 mL) and ether/petroleum ether (1:1, 3×50 mL) and the product (125 g, 75%) was used in the next step without further purification. ^1^H-NMR: 0.91 (t, *J*=6.6 Hz, 6H), 1.29-1.33 (m, 16H), 1.43-1.46 (m, 4H), 1.74-1.79 (m, 4H), 3.92 (t, *J*=6.6 Hz, 4H), 6.84 (s, 4H).

*1,4-bis(Bromomethyl)-2,5-bis(octyloxy)benzene* (**3**): A mixture of 1,4-bis(octyloxy)benzene (**6**, 33.4 g, 0.1 mol), paraformaldehyde (10.8 g, 0.36 mol), HOAc (80 mL) and HBr (40%, 50 mL) was refluxed for 24 hours; after cooling, the precipitate was collected and washed with water. The compound (40 g, 71%) was used for the next step without further purification. ^1^H-NMR: 0.93 (t, *J*=6.6 Hz, 6H), 1.32-1.37 (m, 16H), 1.50-1.53 (m, 4H), 1.84-1.87 (m, 4H), 3.98 (t, *J*=6.6 Hz, 4H), 4.54 (s, 4H), 6.87 (s, 2H). 

*Tetraethyl (2,5-bis(octyloxy)-1,4-phenylene)bis(methylene)diphosphonate* (**4**): A mixture of compound **3** (5.0 g, 10mol) and triethyl phosphite (4.1 g, 0.025 mol) was refluxed for 12 hours, and then the volatiles were stripped off by oil pump. The residue of the desired diphosphonate **4** was used directly. 

*1,4-bis(4'-Nitrostyryl)-2,5-bis(octyloxy)-benzene* (**5**): Under an atmosphere of Ar, t-BuOK (2.47 g, 22 mmol) was added portionwise at 0^o^C to a solution of the residue of **4** mentioned above dissolved in anhydrous THF (30 mL). The mixture was stirred at this temperature for 2 h, then a solution of 4-nitro-benzaldehyde (3.33 g, 22 mmol) in anhydrous THF (10 mL) was added. The reaction mixture was stirred at 0^o^C for 2 hours, and then at 25^o^C for an additional 4 hours. Finally the mixture was quenched with water, the volatiles were evaporated first, then the aqueous phase was extracted with ethyl acetate (3×50 mL), The combined organic extracts were washed with brine (2×10 mL), dried over anhydrous sodium sulfate and evaporated, the product (4.65 g, 74%) after flash column chromatography was obtained as a mixture of *cis* and *trans* isomers **5**.

*(E,E)-1,4-bis(4'-nitrostyryl)-2,5-bis(octyloxy)-benzene* (**5’**): To a solution of the crude compound **5** (4.50 g, 7.1 mmol) in toluene (75 mL) was added I_2_ (90 mg, 0.355 mmol), then the mixture was refluxed for 3 days and monitored by ^1^H-NMR. After completion, the mixture was washed with sodium sulfite, water, brine (10 mL) and dried over anhydrous sodium sulfate. Purification by column chromatography afforded (*E,E*)-1,4-bis(4'-nitrostyryl)-2,5-bis(octyloxy)-benzene (**5’**, 4.27 g, 95%) as a red solid. ^1^H-NMR: 0.88 (t, *J*=6.8 Hz, 6H), 1.27-1.43 (m, 16H), 1.51-1.57 (m, 4H), 1.86-1.91 (m, 4H), 4.07 (t, *J*=6.8 Hz, 4H), 7.12 (s, 2H), 7.21 (d, *J*=16 Hz, 2H), 7.61(d, *J*=8 Hz, 4H), 7.62 (d, *J*=16 Hz, 2H), 8.21(d, *J*=8 Hz, 4H). ^13^C-NMR: 14.56, 23.06, 26.64, 29.65, 29.71, 29.72, 32.12, 69.44, 110.42, 123.78, 126.34, 126.43, 126.59, 127.53, 143.82, 146.03, 150.84.

*(E,E)-1,4-bis(4'-aminostyryl)-2,5-bis(octyloxy)-benzene* (**6**): To a solution of compound **5’** (1.5 g 2.4 mmol) in ethanol (20 mL) and ethyl acetate (20 mL), SnCl_2_ dihydrate (15 g, 80 mmol) was added, and the reaction mixture was refluxed for 8 hours. After completion, water (50 mL) was added and the reaction mixture extracted with ethyl acetate (3×50 mL). The combined organic extracts were washed successively with water and brine, dried over anhydrous sodium sulfate and evaporated, After purification by column chromatography to afford (*E,E*)-1,4-bis(4'-aminostyryl)-2,5-bis(octyloxy)-benzene **6** (1.12g, 83%) as red solid. ^1^H-NMR: 0.88 (t, *J*=7.2 Hz, 6H), 1.27-1.43 (m, 16H), 1.51-1.57 (m, 4H), 1.86-1.91 (m, 4H), 3.74 (s, 4H), 4.03 (t, *J*=6.4 Hz, 4H), 6.65(dt, *J*=8.4 Hz, *J*=2 Hz, 4H), 7.03 (d, *J*=16.8 Hz, 2H), 7.07 (s, 2H), 7.26 (d, *J*=16.8 Hz, 2H), 7.33(dt, *J*=8.4 Hz, *J*=2 Hz, 4H); ^13^C-NMR: 14.60, 23.09, 23.66, 29.67, 29.78, 29.88, 32.15, 69.63, 110.03, 114.90, 119.59, 126.40, 127.30, 127.98, 128.38, 145.33, 150.24.

*(E,E)-1,4-bis(4'-Acetamidostyryl)-2,5-bis(octyloxy)-benzene* (**7**): To a solution of compound **6** (568 mg, 1 mmol), Et_3_N (1 mL) and DMAP (12 mg, 1 mmol) in CH_2_Cl_2_ (10 mL), AcCl (200 mg, 2.5 mmol) was added and the reaction mixture was stirred at room temperature and monitored by TLC. After completion of the reaction, the mixture was quenched with water and extracted with CH_2_Cl_2_ (3×30 mL), then the combined organic extracts were washed successively with water, brine, dried over anhydrous sodium sulfate and evaporated, purification by column chromatography afforded compound **7** (626 mg, 96%) as a red solid. ^1^H-NMR: 0.83 (t, *J*=7.2 Hz, 6H), 1.17-1.38 (m, 16H), 1.57-1.63 (m, 4H), 1.86-1.91 (m, 4H), 2.05 (s, 6H), 3.98 (t, *J*=6.4 Hz, 4H), 6.95(s, 2H), 7.42 (d, *J*=8.4 Hz, 4H), 7.43 (d, *J*=16 Hz, 2H), 7.56 (d, *J*=16 Hz, 2H), 7.64(d, *J*=8.4 Hz, 4H), 9.99(s, 2H); ^13^C-NMR: 14.62, 23.19, 23.64, 24.11, 29.67, 29.78, 29.88, 32.15, 69.67, 110.17, 115.10, 120.35, 126.58, 127.33, 127.98, 128.61, 145.96, 151.82, 168.91.

## Conclusions

In summary, we have designed and synthesized a new diamino substituted Oligophenylvinyl chromophore (*E,E*)-1,4-bis(4'-aminostyryl)-2,5-bis(octyloxy)-benzene (**6**) and its derivative (*E,E*)-1,4-bis(4'-acetamidostyryl)-2,5-bis(octyloxy)-benzene (**7**). At the same time, we have examined the HOMO and the LUMO levels after theoretical calculations. Further application is in progress.
